# Talking about desire to die: Talking past each other? A framework analysis of interview triads with patients, informal caregivers, and health professionals

**DOI:** 10.1017/S1478951524002104

**Published:** 2025-03-24

**Authors:** Kathleen Boström, Thomas Dojan, Thessa Thölking, Leonie Gehrke, Carolin Rosendahl, Raymond Voltz, Kerstin Kremeike

**Affiliations:** 1 Faculty of Medicine and University Hospital, Department of Palliative Medicine, University of Cologne, Cologne, Germany; 2 Department of Anesthesiology, Pain and Palliative Medicine, Radboud University Medical Center, Nijmegen, The Netherlands; 3 Faculty of Medicine and University Hospital, Center for Integrated Oncology Aachen Bonn Cologne Duesseldorf (CIO ABCD), University of Cologne, Cologne, Germany; 4 Faculty of Medicine and University Hospital, Center for Health Services Research, University of Cologne, Cologne, Germany

**Keywords:** Desire to die, palliative care, communication, death talk, framework analysis

## Abstract

**Objectives:**

Up to 40% of seriously ill patients develop a (temporary) desire to die which can lead to requests for assisted dying. Health professionals often feel uncertain about addressing these topics, while informal caregivers may feel guilty and left out. Open and respectful communication proves beneficial. It remains unclear how this communication ideal realizes within the lived experience of all 3 parties. Therefore, we conducted in-depth analysis of communication strategies about desire to die from triangulated perspectives of patients, informal caregivers, and health professionals.

**Methods:**

We conducted semi-structured interviews with purposefully sampled triads consisting of seriously ill patients, their respective informal caregivers and health professionals. Interviews were part of the qualitative evaluation of a 3-phase mixed-methods study on the effects of communication about desire to die on seriously ill patients. We followed a framework analysis approach to build communication types.

**Results:**

From the *N* = 13 patients, 54% suffered from oncological diseases. Health professionals (*N* = 13) were multiprofessional. Informal caregivers (*N* = 13) were partners, children, or another relation. All in all, we conducted *N* = 14 interview triads (*n* = 3 incomplete; *N* = 39 individual interviews).

Four key themes emerged from analysis: (a) how open communication was perceived, (b) whether participants reported shared reality, (c) how they talked about death, and (d) their communication strategies.

Ultimately, 3 communication types were inductively derived at from these key themes. Type 1 “Between the Lines,” type 2 “Past each Other” and type 3 “Matter of Fact” show differing expressions on the key themes, especially on (b) shared reality. Specific type characteristics produce suggestions for health professionals’ communicative practice.

**Significance of results:**

Awareness of typical communication strategies is necessary to foresee potential pitfalls such as loss of information or acting on unchecked assumptions. To reduce distress and increase information flow, health professionals should actively approach informal caregivers for desire to die conversations.

## Background

In the face of life-threatening illness, patients frequently develop a desire to die – an existential experience involving physical, psychological, social, and spiritual aspects. Of those, 12–45% of patients express temporal and 10–18% persistent desire to die (Chochinov et al. 1995; Wilson et al. 2016). Different definitions of desire to die are used to capture the complex phenomenon (Balaguer et al. 2016; Kremeike et al. 2021). We apply a broad understanding that allows for a range of forms, backgrounds, meanings, and functions (Kremeike et al. 2021) and a simultaneous will to live (Voltz et al. 2010). As desire to die is prone to change, we propose the conceptualization along a continuum of increasing suicidal pressure: from acceptance of death or satiety of life to latent or even acute suicidality (Kremeike et al. 2021). The latter can also find expression in the wish for hastened death (Balaguer et al. 2016) or suicidality and wishes for assisted dying (Rodin et al. 2009).

Health professionals are recommended to address desire to die with their patients (German Guideline Programme in Oncology 2020). A proactive approach in an atmosphere of openness, interest, and respect for patients’ thoughts, experiences, and (planned) actions is necessary (Kremeike et al. 2021). If carried out by trained health professionals, desire to die conversations do not harm patients but tend to alleviate depressiveness (Porta-Sales et al. 2019; Voltz et al. 2022).

Ideally, palliative care involves strong therapeutic alliances and shared decision-making between health professionals and patients (Kuosmanen et al. 2021; Thomas et al. 2021), with informal caregivers as important stakeholders. This multiperspectivity likely plays a crucial role concerning desire to die conversations in palliative care. While it may enable better care, it also holds potential for conflicts or misunderstandings: divergent understandings of vital information such as the palliative prognosis are common (Jacobsen et al. 2013). Remaining taboos surrounding death and dying can render the topic unspeakable (Collins et al. 2018a) and may foster denial (Gerber et al. 2020). As humans have a fundamental need for shared reality (Echterhoff et al. 2009), failing to create commonality through communication can cause pain and add to the experience of loneliness in terminal illness (Kang 2021).

Severely ill patients wish for end-of-life conversations with their health professionals (Harding et al. 2013). However, they rather speak with informal caregivers than professionals about suicidal ideation (Lindner et al. 2014) and tend not to address challenging topics like desire to die on their own as to not be a burden (Macmillan Cancer Support 2017).

Health professionals report high levels of uncertainty regarding desire to die conversations (Udo et al. 2014). They also fear to trigger latent suicidality by asking related questions (Allan and Allan 2019), even though asking about suicidality holds no iatrogenic risk (DeCou and Schumann 2018). Therefore, there is a need for specific trainings (Galushko et al. 2016). A multiprofessional training on dealing with desire to die showed increasing levels of health professional confidence thereafter (Boström et al. 2022).

Relatives that operate as informal care providers often know a lot about the patients’ needs and are potentially vital allies in care provision (Fridriksdottir et al. 2006). At the same time, they require support when the patient they are related to desires to die (Metselaar et al. 2019).

Several questions arise when negotiating the topic of desire to die in these interrelationships: Who is included in conversations about desire to die and to what extent? What are contents and potential communicative strategies when talking about desire to die between patients, health professionals, and informal caregivers? What are potential functions of differences in communication styles? And how can health professionals best approach desire to die conversations with patients and informal caregivers? Taking all these aspects into consideration, we aim to explore in what ways patients, health professionals, and informal caregivers experience desire to die conversations and what communication types emerge within these triads.

## Methods

The presented interview data stems from phase 3 of a mixed-methods study aiming to consent a clinical guideline on dealing with desire to die (phase 1), train health professionals in using the guideline (phase 2), and evaluate the effects of a proactive guideline-informed desire to die conversations on severely ill patients, their informal caregivers and health professionals (phase 3) (Kremeike et al. 2018). The study was registered in the German Clinical Trials Register (DRKS00012988; registration date: 27.9.2017).

After the clinical guideline was consented (Kremeike et al. 2020), health professionals participated in a training course based thereon (February 2018–January 2020) (Boström et al. 2022). Trained health professionals recruited suitable patients for an open and proactive conversation on desire to die (April 2018 and March 2020). For an analysis of conversation contents, refer to Boström et al. (2022). Following a quantitative evaluation of the conversation effects on patients (Voltz et al. 2022), a subsample of patients, their health professionals, and a relative were invited by the research team to participate in individual interviews for qualitative evaluation (May 2019–January 2020).

**Figure 1.**
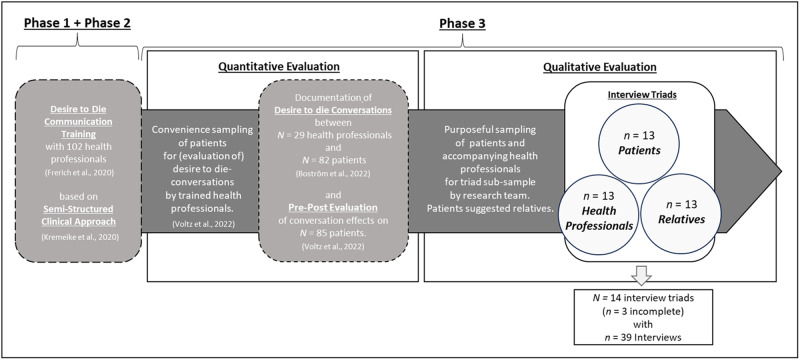
Study procedure with respective sampling strategies for each phase, adapted from Boström et al. (2022).

This article presents the results from this qualitative evaluation of desire to die conversations. For contextualization of the presented interview data within our bigger study and the respective sampling process (Kremeike et al. 2018), see Fig. 1.

### Sample

Sampling in phase 3 of our mixed-methods study (Voltz et al. 2022) took place in a 2-step process. We quantitatively evaluated our desire to die conversations with a larger patient sample and then conducted qualitative interviews with a subsample of patients, health professionals, and relatives derived thereof.

1. Large patient sample for quantitative evaluation (Voltz et al. 2022): We asked health professionals previously trained in dealing with desire to die in to recruit patients following a convenience sampling strategy. Patients were eligible if they (i) had a life expectancy between 3–12 months (estimated by surprise question (White et al. 2017)), (ii) were aged ≥18 years, and (iii) had sufficient cognitive ability and German language skills (Voltz et al. 2022).
2. Subsample for qualitative evaluation: After completion of in-person quantitative data collection, a selection of patients was chosen based on researcher impressions. Following a purposeful sampling strategy, patients were aimed to represent a variety of ages, genders, diagnoses, and care settings, but also insightful experiences. If patients agreed to participate, they were asked to suggest a relative (a person that plays a significant role in their life regardless of family relationship) for an interview. The patients’ health professional was also invited. Interviews were held individually.

To minimize bias, a cover story communicated the study topic to patients and informal caregivers as “end-of-life communication.” Each set of associated patient, health professional and relative interviews formed a triad. We use the term “triad” even in cases where 1 member is missing. Complete and incomplete triads were analyzed together, as they included relevant information on the absent party. To all 2-people-relationships within the triads (patient–relative, patient–health professional, and health professional–relative) we refer as “dyads.”

### Data collection

Interviews were conducted individually at a time and place chosen by each interviewee. Four female researchers (KB, LG, CR, KK) with backgrounds in psychology, nursing, speech therapy, and physiotherapy conducted the interviews following a semi-structured guideline (see Appendix 1). All interviews were audio-recorded and transcribed verbatim. Sociodemographic data was collected using a brief questionnaire.

### Data analysis

Three female (KB, KK, TT) and one male researcher (TD) coded, analyzed, and discussed data. KB and TD have backgrounds in psychology, KK is a physiotherapist and social scientist, and TT a physician and ethicist. All steps of data analysis were conducted using the qualitative data analysis software MAXQDA 2020 (VERBI Software 2019).

**Figure 2.**
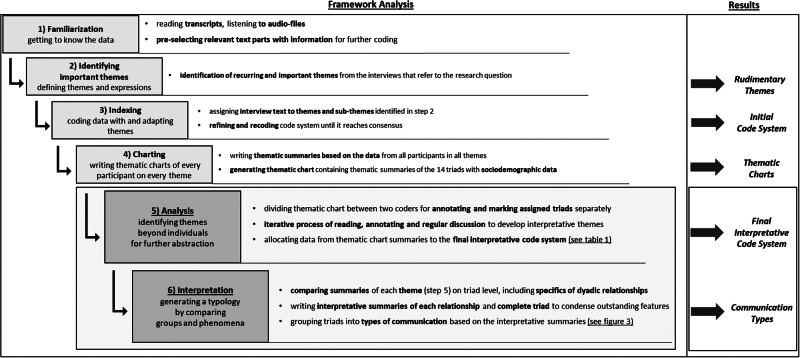
Six step process of framework analysis according to Ritchie and Lewis (2005).

We chose framework analysis according to Ritchie and Lewis (2005) to analyze the interview triads with the aim of generating a communication typology. This method provides the opportunity to manage large sets of qualitative data as well as a dynamic approach to develop a framework from “both a priori issues and emergent data driven themes” (Parkinson et al. 2016). It follows a structuring as well as interpretative approach and lets researchers relate data to existing theories or phenomena. As participants did not strictly differentiate between desire to die conversations and death talk, we included information on both in our analysis. However, if participants reported on persons other than triad members (e.g. further relatives or patients), this information was excluded. For the entirety of the 6-step data analysis process please see Fig. 2.

**Figure 3.**
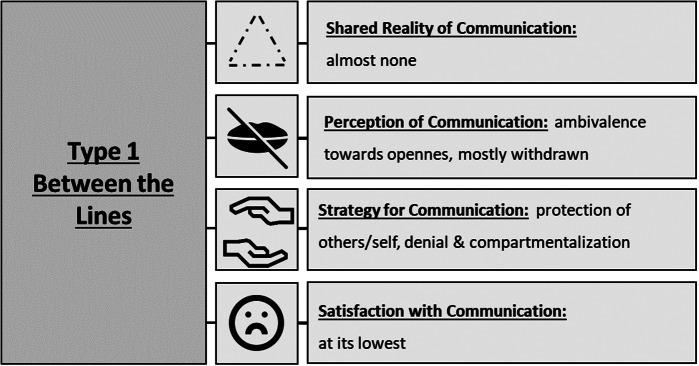
Expressions of key themes in type 1 “between the lines” that describe communication about death and desire to die in the particular triads of patients, health professionals, and informal caregivers.

**Figure 4.**
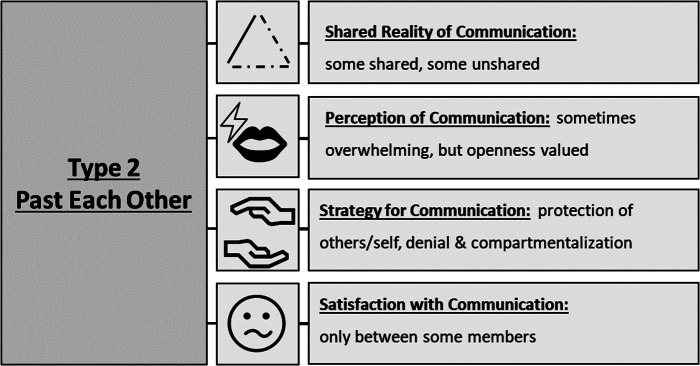
Expressions of key themes in type 2 “past each other” that describe communication about death and desire to die in the particular triads of patients, health professionals and informal caregivers.

**Figure 5.**
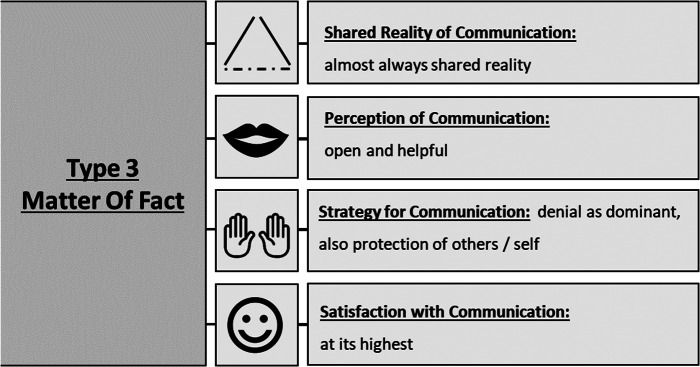
Expressions of key themes in type 3 “matter of fact” that describe communication about death and desire to die in the particular triads of patients, health professionals and informal caregivers.

*All 6 steps of the Framework Analysis process. Steps 1–4 represent preparing analysis steps with steps 5 and 6 highlighted as their results are reported in this paper. For results of step 5, see table in appendix, for results of step 6, see* Figs. 3–5.

Four overarching key themes with 12 subthemes resulted as our final interpretative code system from interpretative analysis in step 5: (1) *How was communication about death and desire to die perceived?*, (2) *Did conversation partners share a reality?*, (3) *By what conversation content was death made understandable?*, and (4) *What strategies are used to communicate about death and desire to die?* (see Table 1).

**Table 1.** Interpretative key themes and subthemes (analysis step 5) used as a basis for building typology

Key themes and subthemes	Definition	Quote
1. How was the communicationperceived? 1.1 Open and helpful 1.2 Open and ambivalent 1.3 Open and overwhelming 1.4 Withdrawn and hindering	Talk about death or desire to die was more or less open. Openness was perceived as helpful, ambivalent, or overwhelming. Withdrawal was usually considered hindering.	*“It was relieving [to talk about the option of assisted suicide with my son]. As if there was a door that was already open, that I didn’t have to push open.”* (Patient, Triad 2)
2. Did conversation partnersshare a reality? 2.1 Shared 2.2 Unshared	Shared reality: similar perception of communication content, atmosphere and each other. Unshared reality: unrelated or paradox perceptions of the same situation.	*“He is my partner after all. He knows me inside out. We talk very intensively and often deeply.”* (Patient, Triad 4) *“That’s very difficult with my wife. She rarely wants to talk about the disease (…). I can’t get through to her anymore.”* (Relative, Triad 4)
3. How was death talkedabout? 3.1 Factual topics 3.2 Existential topics	Factual: e.g. practices like funeral wishes or advance care directives. Existential: e.g. fears or spiritual convictions. Both are not mutually exclusive.	“*I asked her if she was not at all afraid of the medical challenges, of pain, of whatever kinds of symptoms and complaints? She said ‘No, because you will take care of that.’ She was more concerned (…) to have people that accompany her spiritually.”* (Health Professional, Triad 12)
4. What communicationstrategies are used? 4.1 Compartmentalization 4.2 Protecting the other/the self 4.3 Denial 4.4 Acknowledgment	Topics were compartmentalized between conversation partners. Information was not shared based on anticipated burden. The end-of-life situation was not talked about or denied. All acknowledge desire to die and necessary actions. Communication strategies are not mutually exclusive.	*“The conversation (…) was okay (…). Except for having to open up too much which I don’t like to. (…) You don’t have to know everything about me just because I’m dying.”* (Patient, Triad 13)

In step 6, we used specific patterns of expressions on these key themes in each triad to inductively generate 3 communication types (see Fig. 2). Not all key themes proved equally suitable to differentiate between types as some did not sufficiently mark differences (e.g. expressions of *By what conversation content was death made understandable?*). To achieve distinct communication types, we focused on 1 meaningful key theme which varied greatly between triads: *Did conversation partners share a reality?* was used as a basis to cluster by type and other key themes enriched type definitions. We then refined types based on expressed discontent about communication on desire to die from the interpretative triad summaries.

## Results

### Sample

Of 33 patients asked to participate, 14 triads were interviewed (May 2019– February 2020). Three were incomplete: 1 missed a patient (died before interview date), 1 a relative (withdrew due to overburdening), and 1 a health professional (already interviewed before). Therefore, *N* = 39 individual interviews were conducted in total. On average, patient triad interviews took place 103 ± 73 days after the desire to die conversation. There is a large range of time past between desire to die conversation and interviews, since recruitment for qualitative interview evaluation did only start after quantitative evaluation was completed for most patients. Depending on time of entry into the quantitative evaluation, patients had a longer or shorter period between completing quantitative evaluation and being recruited for qualitative interviews. For triad characteristics, see Table 2.

**Table 2.** Participant characteristics

		Patients (*n* = 13)	Health professionals (*n* = 13)	Informal caregivers (*n* = 13)
Age (mean + standard deviation)		68 ± 11	47 ± 11	58 ± 14
Gender	Female	10 (77%)	10 (77%)	8 (62%)
	Male	3 (23%)	3 (23%)	5 (38%)
Characteristics		**Diagnosis** Oncological disease: 7 (54%) Geriatric multimorbidity: 3 (23%) Chronic Obstructive Pulmonary Disease (COPD): 2 (15%) Neurological disease: 1 (8%)	**Profession** Physician: 5 (39%) Nurse: 2 (15%) Social worker: 2 (15%) Other**: 4 (31%)	**Relation** Partner: 5 (39%) Child: 4 (31%) Friend: 2 (15%) Other family member: 2 (15%)***
		Desire to die present* Present: 4 (31%), namely Acceptance of death Desire to die Wish to hasten death Wish for assisted dying Not present: 9 (69%)	**Address of desire to die** Proactive (by health professional): 8 (62%) Reactive (by patient): 5 (38%)	
Education	Baccalaureate	4 (31%)	12 (92%)	6 (46%)
	Higher secondary school	3 (23%)	1 (8%)	5 (38%)
	Lower secondary school	6 (46%)		2 (15%)
Nationality	German	11 (85%)	12 (92%)	12 (92%)
	Other	2 (15%)	1 (8%)	/
	Missing data	/	/	1 (8%)
Duration of interview (minutes)	Mean	49 ± 39	48 ± 20	38 ± 13
	Range	20 − 180	18 − 82	23 − 60
Days between conversation and interview	Mean	98 ± 75	111 ± 75	100 ± 67
	Range	33 − 300	33 − 299	33 − 315
Interview setting		Home: 7 (54%) Residential care facility: 4 (31%) Hospice & Hospital: 1 (8%) each	Home: 2 (15%) Work environment: 11 (85%)	Home: 10 (77%) Other****: 3 (23%)

* as judged and documented by the health professional

** psychologist, nondenominational chaplain, hospice coordinator, speech therapist;

*** daughter in law, niece;

**** work environment, research team office

All health professionals had addressed desire to die with their patient (Boström et al. 2022), but only 4 of the 13 interviewed patients recalled such a conversation (triads 2, 3, 4, 7). Others either did not recall a desire to die conversation at all (triads 5, 10, 8, 9) or remembered it vastly different than their health professional (triads 11, 12, 1, 14, 3). Only in 1 triad the relative was present during the desire to die conversation (triad 1). Informal caregivers and health professionals rarely spoke about desire to die (except triad 1 and 10) and in half of the cases, informal caregivers reported no contact with health professionals at all. Only 2 informal caregivers explicitly wished for more inclusion (triads 4, 6). However, contact between health professionals and informal caregivers might be beneficial: some informal caregivers knew vital information about a patients’ plan for assisted suicide (triads 5, 13) the health professional was not aware of while others reported to suffer from feeling left out by the patient (triads 2, 10).

### Types of communication within triads

Triads differed in their expressions on the interpretative key themes (see Table 1), particularly regarding *Did conversation partners share a reality?*. Thereby, we were able to inductively generate 3 types of communication: *Between the Lines, Past Each Other*, and *Matter Of Fact* (see Figs. 3–5) which, to our knowledge, have not been described elsewhere. Their definitions were enriched with additional details from the 4 other key themes. For a complete list of all triad summaries and their expressions on all 4 key themes according to type, see Appendix 2.

Type 1 – Between the Lines

*He always says: I’m fine. And when I asked, he said to me: Don’t always ask, I feel like shit.* (Relative about patient, triad 6)

Type 1 was found in 5 triads (triads 2, 4, 5, 6, 10). Triad participants **mostly did not share a reality** of the desire to die conversation or death talk. Although often said to be helpful in principle, **openness** about desire to die is experienced as **overwhelming** by members of this type, yet they perceive each other’s **withdrawal as hindering for their communication**. Withdrawal conjured accusations regarding their reasons for being withdrawn, hinting at enmeshed social relations. Their communicative **strategies** reflect that: some chose to keep to themselves **to protect the other or themselves** from burden or unwanted consequences. A few **compartmentalize** and constrict flow of information according to assigned roles (e.g. health professional for medical, relative for personal concerns), while others **deny** the severity of the situation. Patients and informal caregivers of this type report the **lowest satisfaction** due to miscommunication and unmet communication needs, e.g. in triad 4 a husband laments how his wife stopped opening up to him while the wife names him as her most intimate familiar. Death talk mainly focuses on **factual** aspects, yet sometimes **existential** matters emerge.

Type 2 – Past Each Other

*He always tries to be totally helpful and do everything (…). But really with the matter [of the end-of-life], I’m not sure …* (Patient about relative, triad 11)

This type consists of 4 triads (1, 11, 12, 13). Comparing perceptions of desire to die conversations shows a mixed picture. While participants of **some triads report shared reality**, others report **incompatible accounts**. For example, the health professional from triad 1 reported an emotionally challenging educational conversation on the legal possibilities of assisted dying while patient and relative still wrongly believed it to be illegal afterward. Regarding dyadic relations, all **value open communication**, but employ different strategies to achieve it. In their **strategies** to communicate about desire to die or death, most triads engage to a similar degree in **compartmentalization and protection of the other**. Compartmentalization took place based on topic (e.g. spirituality shared with a partner and medical care in the hands of the oncologist, triad 12). Sometimes, this leads to loss of important information. **Satisfaction varies** between individual triad members. While the focus on **factual** aspects is high, the *Past Each Other*-type often address **existential** matters as well.

Type 3 – Matter of Fact

*We talked about symptoms of illness, but we didn’t talk about death. Of course, [about] health care, you know.* (Health professional about relative, triad 9)

This type contains 5 triads (3, 7, 8, 9, 14). Triad participants **all seem to share a reality** on the desire to die conversation, yet often, there either is no desire to die, or patients and informal caregivers report no further need to talk about it. Conversations were perceived as mostly **open and helpful**. In their communicative **strategies**, some fall back on **compartmentalization** or attempt to **protect others**. However, most triad parties show discrepancies in narratives and sometimes direct accusations, hinting at **denial** in dealing with desire to die (triads 8, 9, 14). Triad participants of the 3rd type report **high satisfaction**. Regarding content, triad participants of the *Matter of fact*-type share a focus on **factual** aspects of end-of-life care characterized by a professional attitude. For this type, death talk equates organizing care, e.g. by initiating advance care planning or funeral planning. Therefore, only health professionals of this type acknowledge that the desire to die conversation might not have been perceived as such. The contact between informal caregivers and health professionals (3 of 5 without contact) is remarkably rare.

## Discussion

Talking openly about death and desire to die can offer clarity and emotional relief but also has potential for miscommunication and distress. By exploring such communication in interview triads with patients, health professionals, and informal caregivers, we found 4 interpretative key themes: how *open communication was received*, whether *reality was shared*, what type of *content made death understandable*, and what *communication strategies* were used. From these key themes, we inductively established 3 communication types: *Between the Lines, Past Each Other*, and *Matter of Fact*. Health professionals in are well advised to use different communicative approaches based on the types’ characteristics.

### What the communication types can teach us

Although the unquestionable detection of our types in clinical praxis is difficult, knowledge about them may increase health professionals’ awareness what kind of engagement is required: The *Between the Lines* Type 1 reports high discontent and a communication perceived as withdrawn. Additionally, patients often reported an unexpressed desire to die. They might therefore be the type in highest need of a proactive approach to desire to die by health professionals (Boström et al. 2022; Voltz et al. 2022). It may also enable to initiate adequate psychosocial support. In type 2, *Past Each Other* parties are interested in open communication, but seem to apply diverging strategies. Here, too, informal caregivers are often left out. In this type, an accompanying approach might serve best, to offer stability and guidance as well as prevent transmission errors. On first sight, the *Matter of Fact* approach of Type 3 seems to make for easy communication. Because desire to die barely seems a burdensome topic in Type 3, there is a danger of overlooking concealed or potential desire to die. Health professionals should be sensible toward patients or informal caregivers who put on a façade which might crumble if the burden of disease increases. Overall, 1 hypothesis could be that triads that focus more on facts rather than emotions report more convergent experiences of the desire to die conversations and death talk than triads that focus on emotions rather than facts.

We do not consider it a problem that we did not identify a type with “perfect” communication (i.e. including health professionals, informal caregivers, and patients, perceived as open and satisfying and resulting in correct transmission of information and completely shared reality). Rather, we suggest that even instances of “failed” communication in our results support the notion of communication as always co-constructed and interpretative: people are simultaneously sender and receiver in a process of mutual influence (du Pré and Foster 2016).

### Inclusion in desire to die conversations: who speaks to whom about what?

Due to our study design, health professionals addressed desire to die proactively (Voltz et al. 2022). Thereby, we assume they provide a space for patients to talk about topics relevant to them, either **existential** or **factual** (Boström et al. 2022). Although working through existential topics at the end-of-life is advised (Granda-Cameron and Houldin 2012), such a process can only be encouraged, not enforced. In patient-relative-dyads factual topics dominated and death was usually talked about through organizing care – informal caregivers’ common communication responsibility (e.g. by keeping track of patient’s medical history, diagnosis and prognosis) (Wittenberg et al. 2017). When oneself or a loved one has a life-limiting illness, focusing on factual topics and planning can help experience self-efficacy instead of powerlessness (Nipp et al. 2017; Wittkowski 2015), without risking the emotional vulnerability of addressing existential fears. Informal caregivers’ end-of-life responsibilities can also be cause for immense suffering – a suffering health professional should address (Wittenberg et al. 2017).

In our study, however, meaningful contact between health professionals and informal caregivers was almost nonexistent – common at the end-of-life (Lind et al. 2011). For some informal caregivers, exclusion caused frustration or hindered information flow (e.g. triads 5, 14). As health professionals systematically underestimate informal caregivers’ needs for information and involvement (Collins et al. 2018b), we suggest to actively offer informal caregivers a part in communication about desire to die.

### Perception of desire to die conversations: what is said and what is understood?

A large proportion of triad members reported divergent experiences of desire to die conversations or death talk in general. One cause might be differing inner states and motivations. In communication, the need for **shared reality** is so strong that people often assume others hold the same inner states as they do without checking (Echterhoff et al. 2009). This might explain health professionals assuming a strong impact of the desire to die conversation on their patients, because it had such a strong impact on them.

Divergent perceptions might also stem from the fact that patients often do not want to recall end-of-life conversations, believing they are not as far advanced in their illness (Almack et al. 2012; Granek et al. 2013). Differing memories of conversations between health professionals and patients are also common in high-emotion settings, e.g. breaking bad news (Toutin-Dias et al. 2018). If we consider desire to die conversations a high-emotion setting, health professionals can resort to known communication concepts to account for stress-induced reduced memory capacity. Thereby, they can increase the probability that their words are understood correctly (Hyer and Covello 2017). Paraphrasing contents at the end of conversation, asking for patients understanding and offering follow-up conversations may foster shared reality in desire to die conversations (Makoul and van Dulmen 2015).

### Conversation strategies: straight to the point or past each other?

Most triad members valued **open conversation** regarding death, dying and desire to die. Within the literature, too, there often seems to be a general consensus that open conversation about death and dying is advisable (Granda-Cameron and Houldin 2012). As openness can also be perceived as overwhelming, patients, informal caregivers, and health professionals utilize different strategies to deal with it.

One strategy and a well-researched psychic mechanism is **denial**, which protects the self against an unbearable, threatening truth by refusal to believe it (Blumenthal-Barby and Ubel 2018). Denial has an important protective function, but can also be harmful, e.g., when patients decide against their own values (Friedrichs 2014). In our findings, participants who most often denied the situation were the most content (see Type 3), but also appeared as the most emotionally disengaged and left out important information (e.g., a patients’ ideas on assisted dying).

A recurring reason for developing a desire to die is the fear of being a burden to others (Gudat et al. 2019; Hatano et al. 2021). In this context, **compartmentalizing** communication and support needs between informal caregivers and health professionals makes sense from a patient perspective. Compartmentalizing information might offer psychological relief; slicing difficult to process information in smaller and easier to digest parts.

Compartmentalization was often used to **protect the other/the self** but is not the same. Often, triad members withheld difficult information or emotions from others based on the assumption that the conversation partner would be overwhelmed or react negatively. This indicates a taboo surrounding palliative care and fear of terminal illnesses (Kirby et al. 2018).

Within triad 1, the patient, health professional and relative all **acknowledged** the existence of the patients’ wish for assisted suicide and the need to act. All were present during the desire to die conversation, but vital information was misunderstood, leaving the patient’s son frustrated. Despite recommendations to integrate informal caregivers into the conversation (Leitlinienprogramm Onkologie 2020), this illustrates that it is no fail-safe solution. Due to psychological barriers or unfitting assumptions (Almack et al. 2012), miscommunication may appear. Here, too, common communication concepts might mitigate such loss of information (Hyer and Covello 2017; Makoul and van Dulmen 2015).

### Strength and limitations

To our knowledge, there is no study from palliative care research that combines perspectives of patients, health professionals, and informal caregivers on the same desire to die conversation. Research on perspectives in palliative care often refers to individuals or dyads (Carrillo et al. 2018; Liljeroos et al. 2021). We suggest that our triadic approach allows a broader insight into desire to die conversations and their surrounding atmosphere. Our sample heterogeneity concerning professions (health professionals), diagnoses (patients), and relations (informal caregivers) also allows a tentative generalization.

However, our findings predate the decision of the German Federal Constitutional Court ruling (medically) assisted dying as legal in February 2020 – 1 month after the last triad interview. The reality of requesting assisted dying may change communication about desire to die, as evidence from Canada suggests (Ho et al. 2021). Moreover, prior study experience may have influenced participants’ answers, despite matching semi-structured interview guidelines. Health professionals underwent desire to die training and initiated the conversation, therefore knowing which situation to reflect on. Patients’ participation under the cover story of “end-of-life communication” may have primed them toward this topic. Moreover, time past between desire to die conversation and interview participation might have contributed to the fact that patients could not recall such a conversation, therefore potentially limiting interpretability. However, those 4 patients who did not recall the desire to die conversation at all were not those with the highest number of days between conversation and interview. Informal caregivers had no prior knowledge about the study. Since data was conducted at 1 time point, we cannot examine the entire communication process. Future research could address this in multi-perspective qualitative studies over several time points since desire to die changes over time and is influenced by felt interconnectedness and external events (van Wijngaarden et al. 2021).

## Conclusions

Desire to die communication is recommended to take place in an atmosphere of respect, interest, and openness (Kremeike et al. 2020; Leitlinienprogramm Onkologie DK 2020). Realizing these recommendations while meeting the psychological complexities of information processing might seem challenging. Yet, our findings allow for a few suggestions for practice. Health professionals should

1. offer to integrate informal caregivers as a resource of information on the patient as well as to assess their potential need for support (see also (Foster et al. 2015)).
2. be aware of own potential misjudgments and not act on assumptions, e.g. by asking their patients’ understanding of facts and situations (Makoul and van Dulmen 2015). Here, interest for and openness toward their patients is imperative.
3. be aware of different communicative coping strategies – their own and those of patients and informal caregivers. Staying present in authentic support is key, as communicative misunderstandings will never be fully eradicated.
4. keep balance between acknowledging types of communication and remaining open for individual communication styles.

These recommendations in mind, our findings offer other valuable insight about the nature of communication about death, dying, and desire to die.

## Supporting information

Boström et al. supplementary material 1 Boström et al. supplementary material

Boström et al. supplementary material 2 Boström et al. supplementary material

Boström et al. supplementary material 3 Boström et al. supplementary material

## Data Availability

The datasets generated and/or analyzed during the current study are not publicly available as participants were assured that their personal data may be viewed only by members of the research team but are available from the corresponding author on reasonable request.

## Supplementary material

The supplementary material for this article can be found at https://doi.org/10.1017/S1478951524002104.
