# Supplementary material for: Talking about desire to die: Talking past each other? A framework analysis of interview triads with patients, informal caregivers, and health professionals
Source: Palliat Support Care. 2025 Mar 24;23:e83. doi: 10.1017/S1478951524002104 (PMC13166705; doi:10.1017/S1478951524002104)
Supplement: Boström et al. supplementary material 1 — Boström et al. supplementary material [file S1478951524002104sup001.docx]

**Appendix 1:** Interim results (Analysis Step 3) consisting of themes and sub-themes later used for writing thematic summaries

| **Theme and Sub-Themes** | **Definition and Quote** |
| --- | --- |
| 1. Content  1.1 Variability in Content  1.2 Background, Functions and Expressions of Desire to die  1.3 Therapy Decisions  1.4 Arranging Patient’s End of Life  1.5 Patient’s lived Life | **Definition:** The theme contains information about what specific aspects surrounding death and desire to die triad members talk about. Content may vary regarding on the situation and conversation partner. It may range from the desire to die itself to decisions of care, the organization of the end of life and looking back on patient’s lived life.  **Quote:** *“[W]e talk a lot and [regarding] the topic of death, I would say I already told him a lot, because it always brings about a kind of security for me, when I finally said something out loud. (…) I want to put [this outfit] on and be buried in this place and this saying is very important, just so you know.’ I always discussed these kind of things openly. (laughs)” (Patient, Triad 9)* |
| 2. Form  2.1 Initiation of DDC  2.2 DDC and other Contact with relatives  2.3 Recognition of DDC  2.4 Context of DDC | **Definition:** Within this theme, participants give information on the form of DDCs such as under which conditions the conversation began, whether it was recognized as a conversation about desire to die and in which context it was held. Another formal aspect is the inclusion or exclusion of relatives in DDCs.  **Quote:** *“But there was a time when she was not doing so well and the diagnosis still had to sink in a bit, where she also said that there were moments when she thought about [her desire to die]. But she also didn't talk to anyone. So I was the first one she expressed it to. Neither to her other doctors nor to her husband, and she also said: ‘No, my husband shouldn't know that either.’“ (Health professional, Triad 11)* |
| 3. Evaluation  3.1 Facilitating Factors  3.2 Challenges  3.3 Impact  3.4 Evaluation of DDC as Intervention | **Definition:** In this theme, participants report on how they evaluate the DDC and how they engaged in it as well as its impact on the time after. This evaluation regards the particular DDC conducted within study context as well as death talk in general.  **Quote:** *I: “How did you personally experience [being confronted with the patients’ desire to die]?”*  *R: “I had to swallow. I said ‘How can he say something like that?’ But I have-, I- I was in a situation where you don't know what to answer. No. I can't just say: ‘Now give me a break, it'll be alright.’ But I also can't say: ‘Yes, maybe it would be better for you.’ I couldn’t have said that. I really couldn’t. No, but as I said, he always says: ‘I'm fine.’ And when I asked, he said to me: ‘Don't always ask, I feel like shit.’ Yes, with these words.” (Informal Caregiver, Triad 6)* |
