# Supplementary material for: Talking about desire to die: Talking past each other? A framework analysis of interview triads with patients, informal caregivers, and health professionals
Source: Palliat Support Care. 2025 Mar 24;23:e83. doi: 10.1017/S1478951524002104 (PMC13166705; doi:10.1017/S1478951524002104)
Supplement: Boström et al. supplementary material 2 — Boström et al. supplementary material [file S1478951524002104sup002.docx]

**Appendix 2** – Summaries of all triads within the three types

| **Type 1 – Between the Lines** | | |
| --- | --- | --- |
| **„Enmeshed”** (2) | | |
| - Only health professional- patient and patient-relative spoke about desire to die - Openness as ambivalent, perceived reticence as hindering - Unshared Reality, except health professional-relative - Focus on manifest & material, except health professional - Protecting the Other / the Self, Denial | | The patient (female, 65, cancer) is ambivalent about her hypothetical desire to die and manifest will to live. She does not want to alarm her relative (female, 65, friend) or health professional (female, 48, physician) with suicidal thoughts, but feels her suffering is not taken seriously by her relative. The relative offers a divergent perspective, implying that the patient is in denial about the gravity of her illness and refuses meaningful conversation. The relative and health professional never met which aligns with health professionals’ strong aversion to enmeshed family dynamics. The health professional describes the relationship with the patient as trusting and open, yet barely reports on content from the desire to die conversation. |
| **“Withdrawn”** (4) | | |
| - Only health professional-patient spoke about desire to die - Openness as helpful, yet relative perceives reticence - Unshared Reality - Focus on manifest & material, except patient - Compartmentalization, Denial, Protecting the Self / Other | The patient (female, 77, cancer) sometimes harbors thoughts about suicide, but never tells anyone. She denies her emotional distress, but reports meaningful and deep conversations only with her relative (male, 79, husband). Although she esteems her relative, he is only consulted for his medical knowledge. Her relative’s perspective diverges: he perceives the patient as withdrawn due to her illness and suspects that she prefers to share existential concerns with her health professional (female, 36, psychologist) instead of him. He barely knows the health professional, but displays distress and wishes to be included. The health professional evaluates the desire to die conversation positively and broadly reflects on the topic from a disengaged professional point of view. | |
| **„Transmission errors”** (5) | | |
| - health professional-patient and patient-relative spoke about desire to die - Openness as helpful, yet relative perceives reticence - Unshared Reality - Focus on manifest & material, except health professional - Compartmentalization, Denial | The patient (female, 77, multimorbid geriatric) appears to be ambivalent or erratic regarding her (previously stated) desire to die but also regarding her memory in general –alcohol abuse may have impaired her cognitive functioning. She denies having a desire to die and does not recall the health professional (female, 44, nurse) at all. The health professionals’ perspective diverges: she knows the patient (and her previous desire to die) well and describes their conversation as lively and open. The patient’s relative (female, 58, daughter in law) knows about the end of life preferences of the patient, but has never spoken to the health professional about this crucial information. Information is shared selectively due to compartmentalization: the health professional sees herself as responsible for the patient, not the relative. | |
| **“Disallowance”** (6) | | |
| - All spoke about desire to die - Openness as helpful and overwhelming, yet relative perceives reticence - Unshared Reality - Focus on manifest & material - Compartmentalization, Protecting the Self / Other | At time of the interview triads, the patient (male, 64, cancer) had already passed. The patient opened up to the health professional (female, 64, general practitioner) that he harbored a hypothetical desire to die, though kept it to himself. In contrast, from his relative’s (female, 76, sister) perspective, the patient appeared uncommunicative and withdrawn. She strongly opposes the possibility that the patient might have harbored a desire to die. The desire to die conversation took place with all parties of the triad. Though everybody agreed to this, the health professional in hindsight regrets having included the relative, whom she deems too protective and believes to be in denial of death. Accordingly, the relative reports that they have not spoken about the patient’s (then approaching) death. | |
| **„Whose suffering?”** (10) | | |
| - Only health professional-patient spoke about desire to die - Openness as helpful and overwhelming, yet relative perceives reticence - Unshared Reality - Focus on manifest & material, except health professional - Protecting the Self / Other | The patient (male, 71, COPD) has not talked with his relative (female, 41, daughter) about death or desire to die. He is ambivalent about his wish to do so: on the one hand, he reports no need to, on the other hand, he reports disappointment that his daughter seems not to take his illness seriously enough. His daughter’s perspective diverges: she experiences her father as emotionally withdrawn which causes her intense frustration. Yet, her own fear of addressing the topics that burden him are hindering to her as well. Even though she works in the same institution as the health professional (female, 60, hospice worker), they never talked. The health professional paints yet another picture: despite having to read between the lines, she experienced the desire to die conversation with the patient (mostly about his biography) as successful. The patient does not recall such death talk with his health professional. She also remembers conversations about the patients’ depression with the relative, although she admits not having lots of time for her. She believes the patient shields his daughter from his suffering. | |

| **Type 2 – Past each Other** | |
| --- | --- |
| **„At cross purposes“** (1) | |
| - All spoke about desire to die - Openness as helpful - Unshared reality by all - Focus on manifest & material, except health professional - Integration of Perspectives | The patient (female, 88, multimorbid geriatric) wants do die and she and her relative (male, 58, son) openly talk about assisted suicide. All parties of the triad value openness and respect regarding the patients’ desire to die. There has been a conversation with the entire triad in which the health professional (male, 57, chaplain) offered spiritual support, but also explained the legality of assisted suicide in Germany. However, their perceptions and evaluations of this conversation diverge: The patient does not remember the content of the conversation at all. Her son strongly disliked the spiritual aspects. Both still believe assisted suicide is not legal in Germany, while the health professional was deeply moved by explaining the topic openly to them. |
| **„Rhetoric of fact”** (11) | |
| - Only health professional-patient spoke about desire to die - Openness as helpful, relative is ambivalent - Unshared Reality, except health professional-relative - Focus on manifest & material, except health professional - Compartmentalization, Protecting the Other / the Self | The patient (72, female, cancer) does not count a conversation about advance care planning with her health professional (36, female, general practitioner) as death talk. She dismisses death as nothing special and prefers to talk about it only regarding end of life care. Her health professional perceived their conversation as more existential in nature and was surprised by her patients’ openness and her hypothetical desire to die – although she went easy on her. Her relative (70, male, husband) is ambivalent about her openness: he values deep conversations and death talk as only fitting for their age, yet is overwhelmed by psychological suffering. The patient admits not being able to talk to him openly. The relative and the health professional are in helpful contact, yet not about desire to die. Both relative and patient compartmentalize serious topics to outside the triad. |
| **“Assigned Roles”** (12) | |
| - health professional- patient and patient-relative spoke about desire to die - Openness as helpful - Unshared Reality, except health professional-relative - Focus on manifest & material and personal & existential - Compartmentalization | The patient (57, female, cancer) clearly sees personal and existential topics within her family and not with her health professional (35, female, specialist physician). Her health professional has responsibility for her physical health. She remembers the desire to die conversation to be about preferred place of death. The health professional reports a contrasting account: the patient talked about her spiritual passing into the world of death. Although all value open conversation, the clear assignment of conversational roles aligns with the divergent perspectives of health professional and patient. This triad lacks an interview with a relative (husband), who was reported to only seldomly be present in medical conversations. |
| **„Too overwhelming“** (13) | |
| - health professional- patient and patient-relative spoke about desire to die - Openness as ambivalent, perceived reticence by health professional - Shared reality by health professional-patient, unshared by health professional- relative - Focus on manifest & material and personal & existential - Compartmentalization, Protecting the other / the self | The patient (female, 60, cancer) dominates to which extent death talk may happen and with whom. She draws a line along her ambivalence regarding the topic and casts her relative and health professional in different roles: it is too intimate to talk about the existential aspects with her health professional (54, female, hospice specialist worker), but her relative (55, male, friend) is deeply involved in funeral or potential plans for assisted suicide plans. Convergently, the health professional perceived her as emotionally shut-off, while her relative welcomes her openness and self-reflection, although it sometimes overwhelms him. The health professional does not recall the sparse contact with the relative. |

| **Type 3 – Matter of Fact** | |
| --- | --- |
| **„Not an issue”** (8) | |
| - Only health professional-patient spoke about desire to die - Openness as helpful by all - Shared Reality, except health professional-patient - Focus on manifest & material - Denial | The patient (76, male, cancer) does not recall a desire to die conversation with his health professional (40, male, specialist physician), but also states no need for one. The health professional describes a desire to die conversation, but only in rather general terms. The patient values open conversations with his relative (72, female, wife) and between her and his health professional. She confirms that perception. Desire to die has never been an issue, all focus on concrete matters of life and care. |
| **„Deeply attached”** (9) | |
| - health professional-patient and patient-relative spoke about desire to die - Openness as helpful, only relative ambivalent - Shared Reality, except health professional-patient - Focus on manifest & material, except health professional - Compartmentalization, Denial, Protecting the Other / the Self | The patient (female, 40, MS) names conversations with her relative (46, male, partner) as her main support. They talk about death mainly in concrete plans for the future, but do not frame it as talking about desire to die – even though her relative recalls such a statement. She values the openness; he is ambivalent about it. However, she also aims to protect him from her burden. The relative and the health professional (31, female, speech therapist) did not talk much yet, as they both see the patient in focus. The health professional talks about the helpful open conversation with the patient, while the patient does not name her health professional as a conversation partner once. |
| **„Concrete support”** (14) | |
| - health professional-patient and patient-relative spoke about desire to die - Openness as helpful, only relative ambivalent - Shared Reality - Focus on manifest & material - Denial, Protecting the Other / the Self | The patient (male, 64, COPD) infrequently harbors a desire to die, considering assisted suicide, palliative sedation or just finding rest. He actively plans for his end-of-life via advance care planning which was also topic of his desire to die conversation with his health professional (48, male, hospice coordinator). The patient did not perceive the desire to die conversation as such, but his health professional suspected that much. They both report a good and easy-going relationship revolving around concrete and manifest topics surrounding end of life care. His relative (53, female, niece) supports the patient with daily life tasks and lends an open ear, even though she knows he holds back with more burdensome topics – which the patient confirms. She has no contact to the health professional, but does not see it as an option either way. Predominantly, all triad members evaluate the open communication intended on concrete matters as helpful. However, whereas the patient considers his niece as a potential help in administering assisted suicide in the future, the health professional and the niece both do not know about these hypothetical plans. |
| **„Division of labor“** (3) | |
| - health professional-patient and patient-relative spoke about desire to die - Openness as helpful - Shared Reality - Focus on manifest & material - Compartmentalization, Protecting the Other / the Self | The patient (female, 69, COPD) does not wish to die, but actively plans a conscious end of life. All members of the triad report that they value and lead open conversations about dying and desire to die, thereby sharing the same perspective regarding the situation. Since the health professional (female, 57, hospice nurse) describes the desire to die conversation as strongly focused on life (rather than death), the patient accordingly barely recalls having spoken about desire to die. The patient consciously compartmentalizes her needs between health professional and relative (female, 43, daughter): her health professional brings emotional security, her relative manages worldly affairs. The health professional and relative never met and do not wish to. |
| **„Professionals“** (7) | |
| - health professional-patient and patient-relative spoke about desire to die - Openness as helpful - Shared Reality - Focus on manifest & material - Compartmentalization, Protecting the Other / the Self | The patient (female, 66, cancer) reports having held a desire to die in the past, though not any longer at time of the interview. When asked by her health professional (has not partaken in the interview triad) about discontinuation of life support measures, she wants her physician to decide in her place. With her family, the patient does not discuss death and dying out of fear of being a burden. Convergently, her relative (female, 35, daughter) states that it is difficult to initiate death talk with her mother, who fends off all attempts. Even as a relative, the daughter (herself a general practitioner) recommends taking a professional distance towards desire to die. Death and dying are experienced as non-negotiable and therefore must be approached “professionally”, thereby withdrawing from being personally affected or responsible for decisions. There was no contact between relative and health professional. |
