# Supplementary material for: Talking about desire to die: Talking past each other? A framework analysis of interview triads with patients, informal caregivers, and health professionals
Source: Palliat Support Care. 2025 Mar 24;23:e83. doi: 10.1017/S1478951524002104 (PMC13166705; doi:10.1017/S1478951524002104)
Supplement: Boström et al. supplementary material 3 — Boström et al. supplementary material [file S1478951524002104sup003.docx]

**Semi-structured guideline for qualitative patient interviews**

| **Introduction**  Conversation between patients (P) and their health professionals (HP) are an essential part of care and support. In the case of patients with severe, incurable diseases, it is likely that very personal topics may be addressed. It is not uncommon for patients' own end-of-life issues to come up. Today I would like to talk to you about how you perceive such conversations. |
| --- |

| **Beginning of Interview**  **Communication with Health Professionals**  *[Name + Function]* | **I am especially interested in your communication with your HP. How do you experience conversations with your HP?** | |
| --- | --- | --- |
| Example  Content  Impact  Relative (R) | - How is a typical conversation with HP like? - What are the topics you and HP talk about? - Is there anything that doesn't come up with HP even though you would like to discuss it? - How do you feel during / after a conversation with HP? - You may hear that VG is talking to R. If so, how do you experience the conversations between VG and R? | |
| **Communication about Desire to Die** | **Some people with incurable illness wish that their life may end sooner or that death may come earlier. Perhaps you talked about this with your HP. If so, how was the conversation for you?** | |
| Proactive Approach  Impact  Process | - What is it like to talk about desire to die with HP? - Is there something particular you remember from that conversation? - Who approached the topic desire to die?   - What was it like to address desire to die with HP?   - What was it like to be addressed by HP about desire to die? - What impact does a conversation about desire to die have on you?   - If present, did your desire to die change after such a conversation? - Did your treatment change after the conversation? | |
| **Relationship with Health Professional** | **How would you describe your relationship with HP in general terms?** | |
| VG – P  VG – R | - - How do you experience HP? (As a HP? As a person?) - What makes HP important to you? - How would you rate your relationship between HP and R? | |
| **Communication with Others** | **HP may not be the only person with whom you talk about potential desire to die. If there are other people, how do you experience such conversations with them?** | |
|  | - What people may that be? (Relatives? Friends? Other HPs?) - What do you notice when you compare these conversations with the ones you have with your HP? | |
| **End of Interview** | **You already addressed many interesting aspects. From my side we would come to the end of our interview.** | |
|  | - Is there something that you would like to add regarding the topic, something, that may be important to you or was not addressed yet? - Is there something you want to know or that remains unclear? - How did you experience the interview? - What was your reason for participating in this interview? | |
| **If not addressed yet** | |  |
|  | - Own medical history - Familiy medical history (e.g. suicides within the family) - Influencing factors on desire to die (relieving / aggravating) - Background information on relatives | |
| **Note of Thanks** | **We would like to thank you very much for your participation.**  **The results from this interview will contribute to improve conversations between patients and their health professionals.** | |

**Semi-structured guideline for qualitative health professional interviews**

**Introduction**

Conversation between patients (P) and their health professionals (HP) are an essential part of care and support. In the case of patients with severe, incurable diseases, it is likely that very personal topics may be addressed. It is not uncommon for patients' own end-of-life issues to come up. Today I would like to talk to you about how you experience such conversations with patients or their relatives.

| **Beginning of Interview**  **Communication with Patient** | **I am especially interested in your conversations about desire to die that took place while you participated in our study. How did you experience these conversations?** |
| --- | --- |
| Content  Impact | - What are the contents of desire to die conversations with your patients? - Which aspects of these conversations do you experience as challenging / pleasant? - How do you feel during or after such conversations with your patients? - How do you experience approaching patients potential desire to die proactively since the beginning of the study? |
| **Communication about Desire to Die** | **You also had a conversation with P. How did you perceive this conversation or possible follow-up conversations?** |
| Self Perception  Proactive Approach | - How do you experience yourself in such a conversation? - How do you think P experienced the conversation? - Is there something particular you remember from that conversation? - In your opinion: What did you manage particularly well? What would you like to have done better? - Who addressed the topic of desire to die? What was that like for you? |
| **Relatives** | **There may also have been conversations with R in which desire to die was an issue.** |
| R – HP  R – P  Generell | - If so, how is it like to talk with R about desire to die? - Does the relationship dynamic allow for a conversation about desire to die? - How do you as a HP experience conversations about the topic of desire to die between P and R? - How do you exerience talking about the topic desire to die with R in general? Compared to conversations with P? |
| **Relationship with Patient / Relative** | **How would you describe your relationship with the P?** |
| HP - P  HP - R | - What impact did the conversation have on your relationship with P? - Why did you recruit P for the study? - How do you experience the relative of P? - How would you describe your relationship with R? - How does Ps and Rs relationship influence your communication with P? |
| **Study Participation** |  |
| Communication Guideline  Study | - Which role did the communication guideline play in your conversation? - How was using the documentation sheet like? - Perhaps something changed for you - either personally or professionally - since study participation. If so, what impact did the study participation have on you? - How did your attitude regarding proactive desire to die change? Todeswünschen? - What was your motivation to participate in the study? |
| **End of Interview** | **You already addressed many interesting aspects. From my side we would come to the end of our interview.** |
|  | - Is there something that you would like to add regarding the topic, something, that may be important to you or was not addressed yet? - Is there something you want to know or that remains unclear? - How did you experience the interview?   What was your reason for participating in this interview? |
| **Note of Thanks** | **We would like to thank you very much for your participation.**  **The results from this interview will contribute to improve conversations between patients and their health professionals.** |

**Semi-structured guideline for qualitative relative interviews**

**Introduction**

Conversation between patients (P) and those who care for them are an essential part of care and support. In the case of patients with severe, incurable diseases, it is likely that very personal topics may be addressed. It is not uncommon for patients' own end-of-life issues to come up. As a relative (R), you experience a lot on the side of the patient (P). Perhaps you are also in direct contact with their health professional (HP). In this context, I would like to talk to you today about your perception of consultative and supporting conversations in palliative care.

| **Beginning of Interview**  **Communication in Care** | **I am especially interested in your conversations with HP. Did you have a chance to talk to HP? If so, how do you experience conversations with HP?** |
| --- | --- |
| R-HP  Perspective on HP-P | - How would you describe your involvement in the treatment of P? - How close or intense would you describe the content between you and HP? - What is it you talk about with HP? - Is there anything that doesn't come up with HP even though you would like to discuss it? - How do you feel during or after conversations with HP? - If you had any Falls es bereits one-to-one conversations with HP: How are they like in comparison to those with P present? - How do you experience the conversations between HP and P if you are present? |
| **Communication about Desire to die** | **Some people with incurable illness wish that their life may end sooner or that death may come earlier. Perhaps you talked about this with the P or the HP. If so, how do you experience talking about desire to die for you?** |
| Example  Perspective on HP-P  R-HP  R-P  In general | - Do you recall a particular conversation? - What do you think of the HP talking to you or the P about desire to die? - In your opinion, how do these conversations affect P?   - If present: How do the conversations influence the desire to die?   How do you experience the communication between HP and P since study participation in general?   - How is it like or would it be like to talk about Ps desire to die with the HP?   - Burden? Own desire to die? - If you ever talked about desire to die with P, how was it like? - What do you experience as helpful and what as Was erleben Sie als hilfreich und was als hindering when talking about desire to die?   With HP? With *P*?   - What is your attitude toward talking about desire to die at all in palliative care?   - With *P*? With *ZG*? |
| **Relationship** | **How would you describe your relationship with HP?** |
| R-HP  Perspective on HP-P  R-P | - - How do you experience HP? (As a HP? As a person?) - How would you rate your relationship between HP and R? How do you experience them both together? - How would you rate the HPs aptitude for conversations about desire to die? - How is your relationship with P? |
| **Communication with Others** | **HP may not be the only person with whom you talk about potential desire to die. If there are other people, how do you experience such conversations with them?** |
|  | - What people may that be? (Relatives? Friends? Other HPs?) - What do you notice when you compare these conversations with the ones you have with HP or P? |
| **End of Interview** | **You already addressed many interesting aspects. From my side we would come to the end of our interview.** |
|  | - Is there something that you would like to add regarding the topic, something, that may be important to you or was not addressed yet? - Is there something you want to know or that remains unclear? - How did you experience the interview?   What was your reason for participating in this interview? |
| **Note of Thanks** | **We would like to thank you very much for your participation.**  **The results from this interview will contribute to improve conversations between patients and their health professionals.** |
